# The prognostic significance of LncRNA BLACAT1 overexpression in various tumors: a meta-analysis

**DOI:** 10.3389/fgene.2024.1362420

**Published:** 2024-03-27

**Authors:** Xuefen Yan, Nana Zhang, Gang Wang, Jiaheng Wang

**Affiliations:** The Quzhou Affiliated Hospital of Wenzhou Medical University, Quzhou People’s Hospital, Quzhou, Zhejiang, China

**Keywords:** cancer, long non-coding RNA BLACAT1, meta-analysis, prognosis, survival

## Abstract

**Objective:**

Recent studies have revealed increasing evidence that the long non-coding RNA bladder cancer associated transcript 1 (LncRNA BLACAT1) plays an essential role in the emergence of different malignancies. This meta-analysis aimed to evaluate the prognostic significance of LncRNA BLACAT1 in various cancers.

**Methods:**

Six electronic databases (PubMed, Embase, Medline, Web of Science, China National Knowledge Infrastructure (CNKI), and the Chinese WanFang database) were comprehensively searched for relevant studies. The analysis of overall survival (OS) and clinicopathological characteristics was conducted.

**Results:**

Nineteen studies with 1,559 patients were eventually eligible to be included in this meta-analysis. High expression level of LncRNA BLACAT1 was identified to be linked with shorter OS (HR: 2.02, 95% CI: 1.66–2.46, *p* < 0.001) and PFS (HR: 2.424, 95% CI: 1.827–3.020, *p* < 0.001) in cancer patients as opposed to low expression levels. Subgroup analysis showed that analysis model (multivariate or univariate), cut-off value (mean or median), sample size (more or fewer than 100), and cancer type had little effect on OS in multiple tumors. Moreover, high LncRNA BLACAT1 expression was associated with positive lymph node metastasis (HR: 2.29, 95% CI: 1.66–3.16, *p* < 0.00001), advanced clinical stage (HR: 2.29, 95% CI: 1.65–3.19, *p* < 0.00001) and worse differentiation status (HR: 0.58, 95% CI: 0.37–0.92, *p* = 0.02), compared to low LncRNA BLACAT1 expression.

**Conclusion:**

The findings highlight that high LncRNA BLACAT1 expression might be detrimental and induce a worse prognosis for cancer patients.

## Introduction

Cancer is a major threat to human health worldwide and brings a heavy economic burden to the world every year. According to Cancer Statistics, approximately 28.4 million cases will be diagnosed in 2040 (an increase of 47% compared to 2020) (Sung et al., 2021). In the United States, approximately 1,918,030 cancer cases were diagnosed, and 609,360 cancer-related deaths were reported, according to the American Cancer Society (Siegel et al., 2022). Despite tremendous progress in the diagnosis and treatment of cancer in recent years, the overall survival rate of cancer patients has not improved significantly. The lack of effective biomarkers to predict prognosis is considered an important reason for poor prognosis in cancer patients.

Long noncoding RNAs (LncRNA) is a type of non-coding RNAs composed of more than 200 nucleic acids (Chen, 2016), that play a crucial role in regulating gene expression at various points in the transcription/translation process. Numerous studies have found that lncRNA plays is essential for the pathogenesis and prognosis of malignancies as well as several biological processes, including the regulation of the proliferation, invasion, and metastasis of cancer (Schmitz et al., 2016). Thus, lncRNA can be used as biomarkers and therapeutic targets for cancer.

Long noncoding RNA bladder cancer associated transcript 1 (LncRNA BLACAT1), also known as linc-UBC1, which was first reported to be located at human chromosome 1q32.1, is 2616 bp in length (He et al., 2013). Many emerging studies demonstrated that BLACAT1 was aberrantly expressed in different cancers, including gastric cancer, glioma, lung cancer, and cervical cancer (Huang et al., 2019; Liu et al., 2019; Ju et al., 2020; Wang et al., 2020). Although LncRNA BLACAT1 expression was associated with poor overall survival and clinicopathological features (Chen et al., 2018; Cheng et al., 2020), the significance of these associations has not been adequately estimated due to the limitation of sample sizes from individual studies. Therefore, we conducted this meta-analysis to explore the prognostic value of LncRNA BLACAT1 in cancer patients.

## Methods

### Search strategy

Potentially literature was searched through PubMed, Embase, Web of Science, Cochrane Library, China National Knowledge Infrastructure (CNKI), and WanFang database. The last literature search was conducted on 5 March 2024. The search terms were as follows: (“long noncoding RNA BLACAT1” OR “lncRNA BLACAT1” OR “BLACAT1” OR “bladder cancer associated transcript 1” OR “linc-UBC1”) AND (“cancer” OR “tumor” OR “carcinoma” OR “neoplasm” OR “neoplasia”). The detailed search strategy was described in Supplementary Table S1. The references of included studies will also be checked to avoid potential omissions.

### Inclusion and exclusion criteria

The inclusion criteria were as follows: 1) retrospective or prospective cohort studies; 2) LncRNA BLACAT1 expression was identified in cancerous tissues and corresponding non-cancerous tissues through qRT-PCR; 3) patients were divided into high LncRNA BLACAT1 expression group and low LncRNA BLACAT1 expression group; 4) reported sufficient data about the relationship between LncRNA BLACAT1 expression level and overall survival (OS), disease-free survival (DFS), or clinicopathologic parameters; 5) reported sufficient data for the assessment of hazard ratios (HRs) and 95% confidence intervals (CIs). The exclusion criteria were as follows: 1) duplicate articles; 2) reviews, letters, conference reports, and animal studies; 3) articles with insufficient data (e.g., without OS, DFS, or clinicopathologic parameters).

### Data extraction and quality assessment

Two researchers searched and evaluated the studies independently according to the inclusion criteria. The following items were extracted: first author, publication year, country or region, cancer type, number of cases, detection method, cut-off value, follow-up months, and analysis method. In addition, clinicopathological parameters including age, gender, tumor size, lymph node metastasis, clinical stage, and distant metastasis were also extracted. The prognostic endpoints included overall survival (OS), progression-free survival (PFS), recurrence-free survival (RFS), and disease-free survival (DFS). HRs and 95%CIs were directly obtained from the univariate or multivariate analysis. If HRs and 95% CIs were not provided, Engauge Digitizer V4.1 software was used to extract HR and 95% CI. The quality of the studies was assessed using the Newcastle–Ottawa Scale (NOS) criteria. Articles with a NOS score ≥6 were considered high-quality; otherwise, they were regarded as low-quality (Stang, 2010). Any disagreements between the two reviewers were resolved by discussion and consensus.

### Statistical analysis

All statistical analyses were performed using Review Manager version 5.4 software (RevMan, The Cochrane Collaboration, Oxford, UK) and Stata 16 software (StataCorp, College Station, TX, USA). Pooled HRs and 95% CIs were used to assess the association between LncRNA BLACAT1 expression and the clinical prognosis of the cancer patients. The inter-study heterogeneity was assessed by I^2^ statistics. If I^2^ ≤ 50% indicated there is no obvious heterogeneity among included studies, a fixed-effects model was used. Otherwise, the random-effects model was used. Sensitivity analysis was also applied to assess the stability of the combined results and to determine the source of any heterogeneity. Egger’s funnel regression test and Begg’s funnel plot were performed to evaluate the publication bias. A two-tailed *p*-value < 0.05 indicated statistically significant.

## Results

### Study search

**FIGURE 1:**
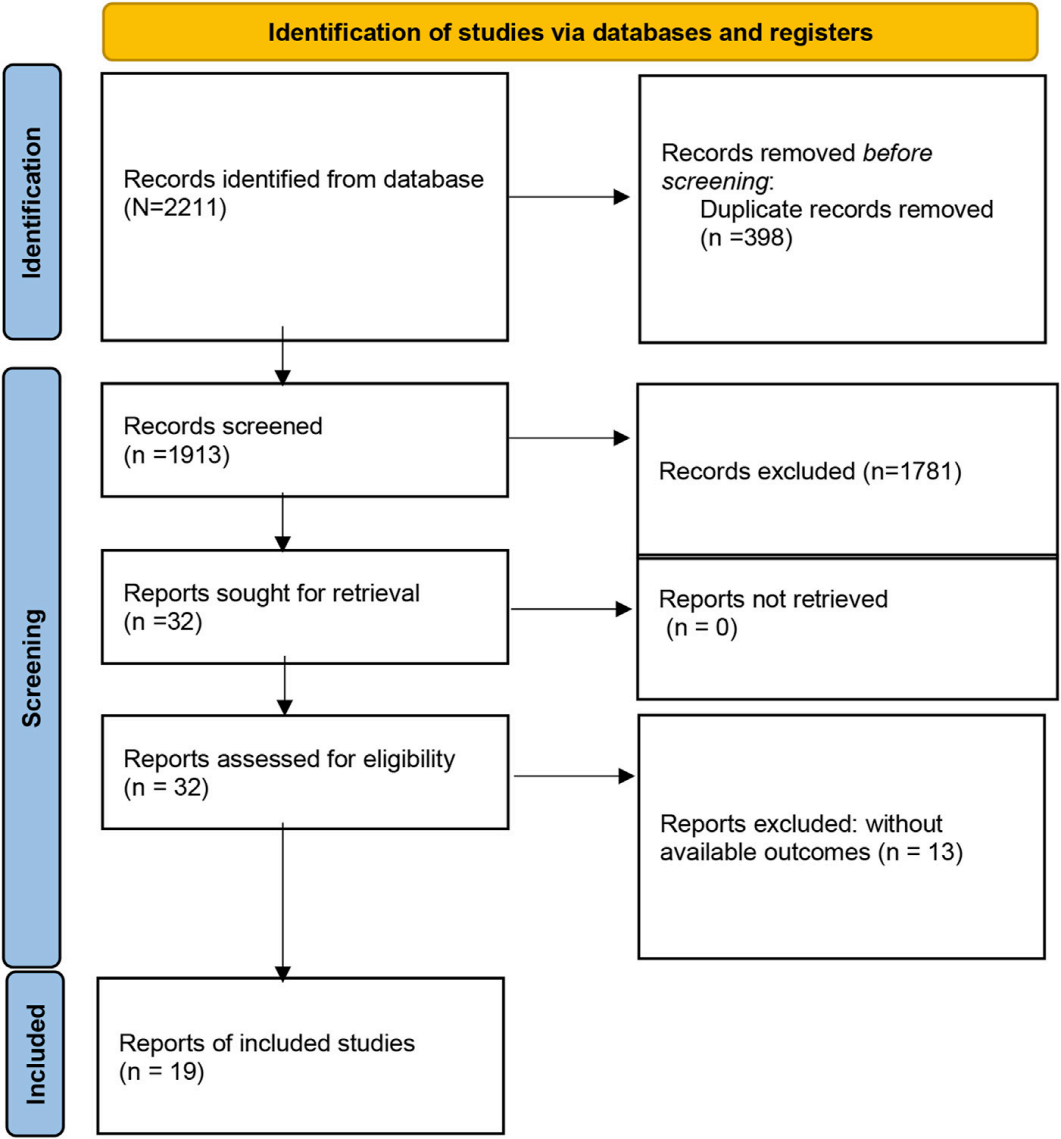
Flow diagram of the study selection process.

A total of 2211 studies were preliminarily retrieved by searching the PubMed, Embase, Medline, Web of Science, CNKI, and Wanfang databases. 298 articles were excluded for duplicates. Of the remaining 1813 papers, 1981 were directly excluded by scanning the titles or abstracts. Then, 32 potentially relevant articles were selected for full-text review, and 13 articles were removed because of inefficient data to estimate HR for further analysis. Finally, nineteen studies comprising 1,559 patients were eligible to be included in this meta-analysis (Figure 1).

### Characteristics of included studies

Detailed characteristics of the included studies were listed in Table 1. Eighteen studies were conducted in China, but one in Germany. The included studies had a wide range of sample sizes, ranging from 45 to 156. The following cancers were investigated: acute myeloid leukemia (AML) (Wu et al., 2020), colorectal cancer (CRC) (Gao et al., 2017; Chen et al., 2020), small-cell lung cancer (SCLC) (Chen et al., 2018), head and neck squamous cell carcinoma (HNSCC) (Gou et al., 2020), gastric cancer (GC) (Hu et al., 2015), osteosarcoma (Dong and Wang, 2019), glioma (Liu et al., 2019; Zhang et al., 2021), breast cancer (BC) (Hu et al., 2019), bladder cancer (BLCA) (He et al., 2013), urothelial carcinoma (UC) (Droop et al., 2017), esophageal squamous cell carcinoma (ESCC) (Niu et al., 2018), cervical cancer (CC) (Wang et al., 2018; Cheng et al., 2020) and non-small cell lung cancer (NSCLC) (Ye et al., 2017; Xu et al., 2019). The LncRNA BLACAT1 expression was detected by qRT-PCR in all studies. The cut-off value for calculating LncRNA BLACAT1 expression was the mean, median, fold change, or Youden’s index, and one study gave no details about the cut-off value. Additionally, all included studies got NOS scores of 7 or more, illustrating their high methodological quality.

**TABLE 1:** The characteristics of the included studies.

Study	Year	Country	Cancer type	Total number	BLACAT1 expression (L/H) (n)	Stage	Sample	Detection method	Cut-off value	Follow-up months	Survival analysis	Analysis model	HR	LCI-UCI	NOS
Cheng HL	2019	China	CC	60	32/28	I–IV	Tissues	qRT-PCR	Youden’s index	60	OS	U	1.62	0.61–4.30	8
Chen R	2020	China	CRC	55	27/28	I–IV	Tissues	qRT-PCR	Mean	60	OS	U	2.33	0.92–5.90	8
Chen WW	2018	China	SCLC	96	53/53	I–IV	Tissues	qRT-PCR	Median	60	OS	M	1.79	1.03–3.08	8
Dong Z	2019	China	Osteosarcoma	82	44/38	I–IV	Tissues	qRT-PCR	Mean	60	OS	U	2.33	0.81–6.67	8
Droop	2017	Germany	UC	106	53/53	I–IV	Tissues	qRT-PCR	Median	60	OS	M	0.95	0.62–1.47	7
Gou CX	2020	China	HNSCC	73	36/37	III–IV	Tissues	qRT-PCR	Median	60	OS	M	2.16	1.18–3.94	8
Gao XF	2017	China	CRC	96	48/48	I–IV	Tissues	qRT-PCR	Median	60	OS	M	2.43	1.09–5.42	8
Hu YR	2015	China	GC	85	53/32	I–IV	Tissues	qRT-PCR	Median	100	OS	U	3.71	1.33–10.40	7
Hu XP	2019	China	BC	72	NA	I–IV	Tissues	qRT-PCR	NA	60	OS	U	1.42	0.65–3.12	7
He W	2013	China	BLCA	102	42/60	I–IV	Tissues	qRT-PCR	fold change of >1.5	60	OS	U	1.69	0.68–4.18	8
Liu J	2019	China	glioma	45	NA	II–IV	Tissues	qRT-PCR	median	60	OS	U	3.06	1.30–7.20	7
Liu NJ	2019	China	glioma	47	NA	I–IV	Tissues	qRT-PCR	median	60	OS	U	1.84	0.54–6.36	7
Niu GC	2018	China	ESCC	50	24/26	I–IV	Tissues	qRT-PCR	median	60	OS	U	2.37	0.83–6.77	8
Su J	2017	China	CRC	48	24/24	I–IV	Tissues	qRT-PCR	median	60	OS	M	1.50	1.32–1.70	8
Wang CH	2018	China	CC	133	65/68	I–IV	Tissues	qRT-PCR	median	60	OS	M	3.93	1.66–7.46	8
Wu XL	2020	China	AML	68	NA	I-IV	Tissues	qRT-PCR	median	40	OS	M	5.33	2.96–12.85	7
Xu R	2019	China	NSCLC	156	78/78	I–IV	Tissues	qRT-PCR	median	60	OS	M	2.08	1.59–2.48	8
Ye JR	2017	China	NSCLC	48	20/28	I–III	Tissues	qRT-PCR	median	48	OS	U	1.57	0.54–4.50	7
Zhang XJ	2021	China	glioma	137	70/67	I–IV	Tissues	qRT-PCR	median	60	OS	M	2.74	1.79–8.23	8

Note: AML, Acute Myeloid Leukemia; CC, Cervical cancer; CRC, Colorectal Cancer; SCLC, small-cell lung cancer; HNSCC, Head and neck squamous cell carcinoma; GC: gastric cancer; BLCA, bladder cancer; UC, urothelial carcinoma; ESCC, esophageal squamous cell carcinoma; NSCLC, non-small cell lung cancer; OS, overall survival; M, multivariate; U, univariate; HR, hazard ratio; LCI, lower confidence interval; UCI, upper confidence interval; NOS, Newcastle-Ottawa Scale; NA, not available.

### The relationship between the LncRNA BLACAT1 expression and survival outcomes

**FIGURE 2:**
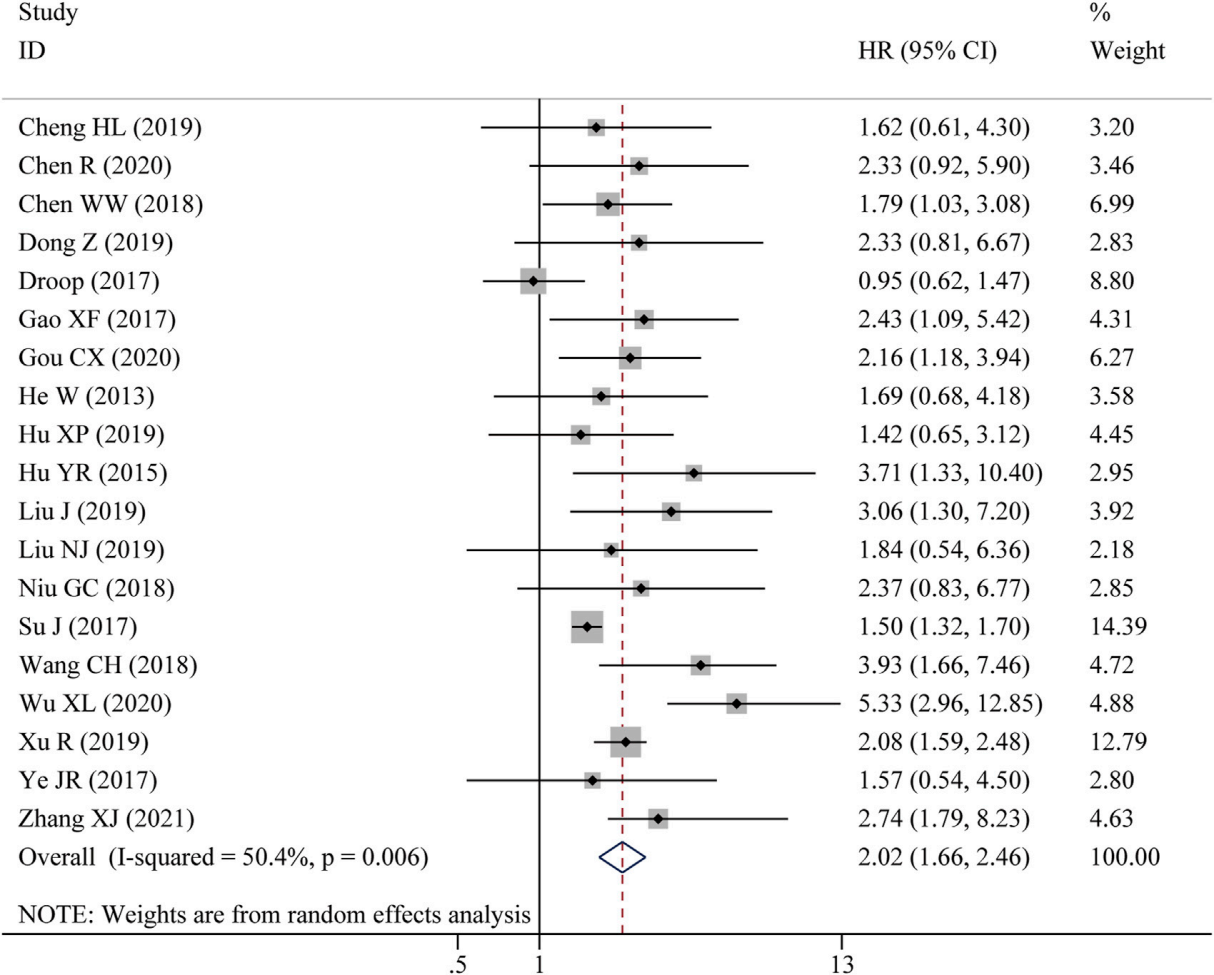
Forest plot shows the association between the LncRNA BLACAT1 expression and overall survival (OS). The rhombus represents the pooled effect, the center of the rhombus represents the pooled effect size, and the width of the rhombus represents the confidence interval. The vertical line represents HR = 1, and any confidence interval crossing this vertical line represents non-statistically significant.

**FIGURE 3:**
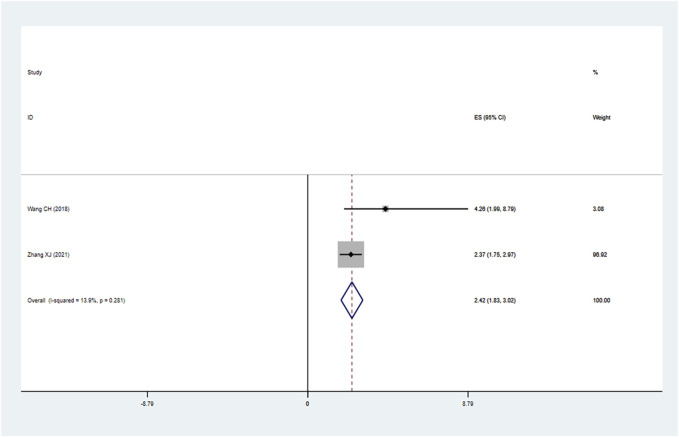
Forest plot shows the association between the LncRNA BLACAT1 expression and progression-free survival (PFS). The rhombus represents the pooled effect, the center of the rhombus represents the pooled effect size, and the width of the rhombus represents the confidence interval. The vertical line represents HR = 1, and any confidence interval crossing this vertical line represents non-statistically significant.

Nine studies reported the OS assessed by multivariate analysis and ten studies reported the OS in the form of a survival curve, therefore, nineteen studies were finally included in the meta-analysis of OS. As shown in Figure 2, due to obvious heterogeneity among included studies (I^2^ = 50.4%, *p* = 0.006), a random-effects model was adopted. The pooled results showed that high LncRNA BLACAT1 expression was significantly correlated with inferior OS (HR: 2.02, 95% CI: 1.66–2.46, *p* < 0.0001), which means the cancer patients with low BLACAT1 expression had a better prognosis. Moreover, two studies reported PFS data. As shown in Figure 3, the pooled results indicated that high LncRNA BLACAT1 expression was related to poor PFS (HR: 2.424, 95% CI: 1.827–3.020, *p* < 0.0001, I^2^ = 13.9%).

As listed in Table 2, subgroup analyses stratified by survival analysis method, cut-off value, sample size, and cancer type were also performed. For the survival analysis method, the HRs of nine studies were calculated using multivariate analysis, and the subgroup analysis showed that LncRNA BLACAT1 expression was significantly associated with the OS (*p* < 0.0001), Similarly, high LncRNA BLACAT1 expression was an unfavorable independent prognostic factor for OS based on univariate analysis (*p* < 0.0001). After stratifying based on cut-off values, the results indicated that no matter how to cut off the expression, there was a distinct association between LncRNA BLACAT1 expression and OS in cancer patients (*p* < 0.0001). After stratifying based on sample size, LncRNA BLACAT1 was found to be a poor prognostic factor in studies with a sample size less than 100 (HR: 1.55, 95% CI: 1.37–1.73) or more than 100 (HR: 1.80, 95 CI: 0.92–2.68). In addition, subgroup analyses based on cancer type were also carried out, and results revealed that LncRNA BLACAT1 expression was significantly associated with poor OS in patients with specific cancers, including colorectal cancer (HR: 1.51, 95% CI: 1.32–1.70, *p* < 0.0001) and lung cancer (HR: 2.02, 95% CI: 1.62–2.42, *p* < 0.0001).

**TABLE 2:** Stratified analyses of the pooled HRs of overall survival by analysis model, cut-off value, sample size, and cancer type.

Subgroup analysis	Included studies	Patients(n)	Pooled HR (95% CI)	*p*	Heterogeneity		Model
					I^2^ (%)	*p*	
Analysis Model							
Multivariate	9	913	2.05 (1.56, 2.70)	<0.0001	74.1	0.000	random
Univariate	10	646	1.83 (1.15, 2.50)	<0.0001	0	0.986	fixed
Cut-off Value							
Mean	2	137	2.33 (0.43, 4.23)	0.016	0	1.000	fixed
Median	13	1,120	1.54 (1.38, 1.69)	<0.0001	36.0	0.088	fixed
Sample Size							
<100	13	857	1.55 (1.37, 1.73)	<0.0001	0	0.965	fixed
≥100	5	634	1.80 (0.92, 2.68)	<0.0001	75.4	0.003	random
Cancer Type							
CC	2	193	2.29 (0.73, 3.84)	0.004	42.4	0.188	fixed
CRC	2	199	1.51 (1.32, 1.70)	<0.0001	0	0.571	fixed
Lung cancer	3	300	2.02 (1.62, 2.42)	<0.0001	0	0.794	fixed
Glioma	3	229	2.53 (0.79, 4.27)	0.004	0	0.837	fixed

### Relationship between LncRNA BLACAT1 overexpression and clinicopathological characteristics

**FIGURE 4:**
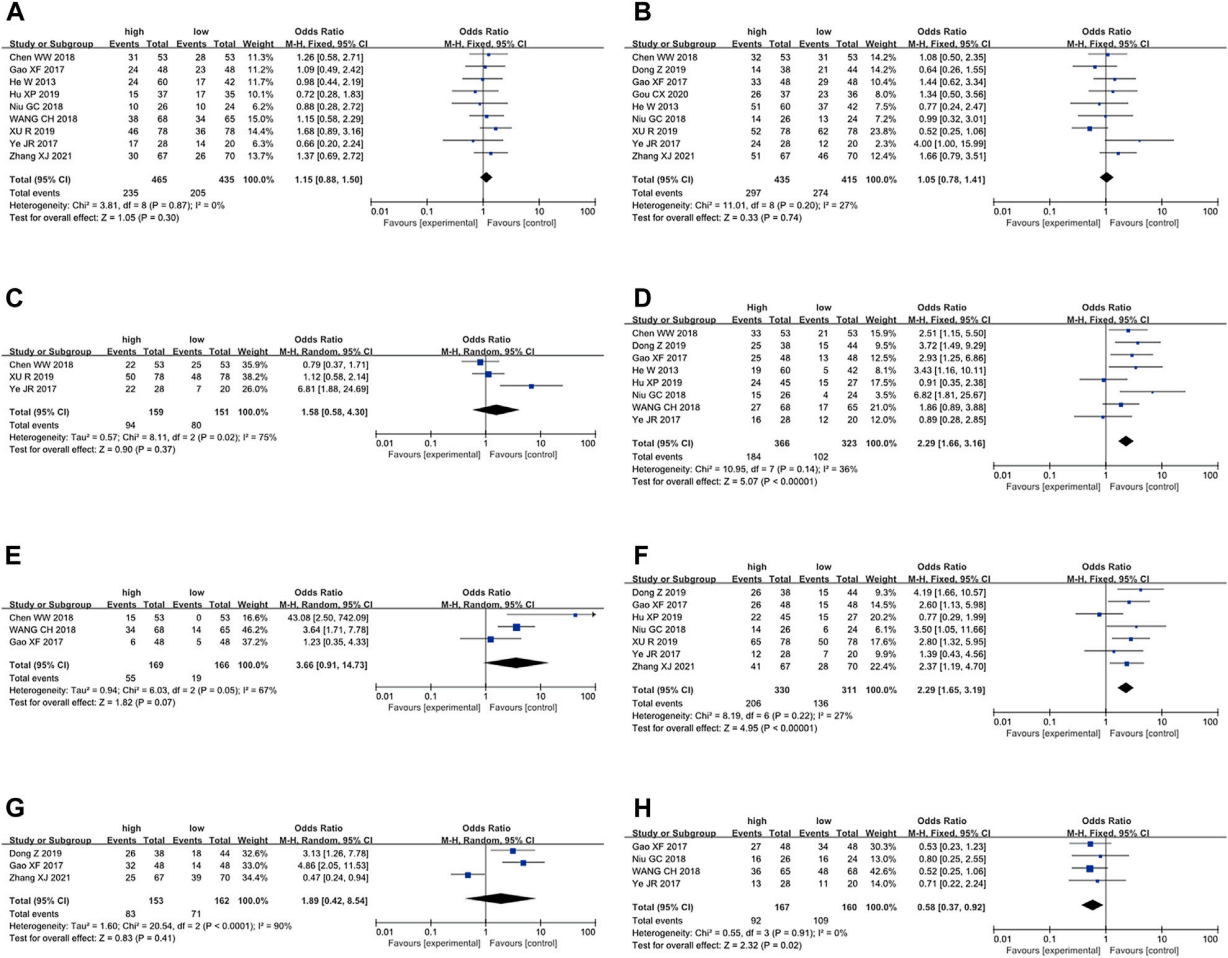
Forest plot shows the association between the LncRNA BLACAT1 expression and clinicopathological characteristics of solid tumors: **(A)** age; **(B)** gender; **(C)** smoking; **(D)** lymph node metastasis; **(E)** distant metastasis; **(F)** clinical stage; **(G)** tumor size; **(H)** differentiation status. The rhombus represents the pooled effect, the center of the rhombus represents the pooled effect size, and the width of the rhombus represents the confidence interval. The vertical line represents HR = 1, and any confidence interval crossing this vertical line represents non-statistically significant.

To further elucidate the clinical implications of LncRNA BLACAT1 overexpression, we examined the association between LncRNA BLACAT1 expression and clinicopathological characteristics (Table 3; Figure 4). The results showed that high LncRNA BLACAT1 expression was significantly associated with positive lymph node metastasis (*p* < 0.00001), advanced clinical stage (*p* < 0.00001) and poor differentiation status (*p* = 0.02). However, there was no discrepancy in age (*p* = 0.30), gender (*p* = 0.74), smoking (*p* = 0.37), tumor size (*p* = 0.41) and distant metastasis (*p* = 0.07).

**TABLE 3:** Correlation between LncRNA BLACAT1 expression and clinicopathological characteristics of solid tumors.

Variables	Included studies	Patients(n)	OR	95% CI	*p*	I^2^^(%)^	Model
Age (old vs. young)	9	900	1.15	0.88–1.50	0.30	0	Fixed
Gender (male vs. female)	9	850	1.05	0.78–1.41	0.74	27	Fixed
Smoking (yes vs. no)	3	310	1.58	0.58–4.30	0.37	75	Random
Lymph node metastasis (yes vs. no)	8	689	2.29	1.66–3.16	<0.00001	36	Fixed
Distant metastasis (yes vs. no)	3	335	3.66	0.91–14.73	0.07	67	Random
Clinical stage (III–IV vs. I–II)	7	641	2.29	1.65–3.19	<0.00001	27	Fixed
Tumor size (cm) (≥5 vs. < 5)	3	315	1.89	0.42–8.64	0.41	90	Random
Differentiation status (well/moderately vs. poorly)	4	327	0.58	0.37–0.92	0.02	0	Fixed

### Publication bias

**FIGURE 5:**
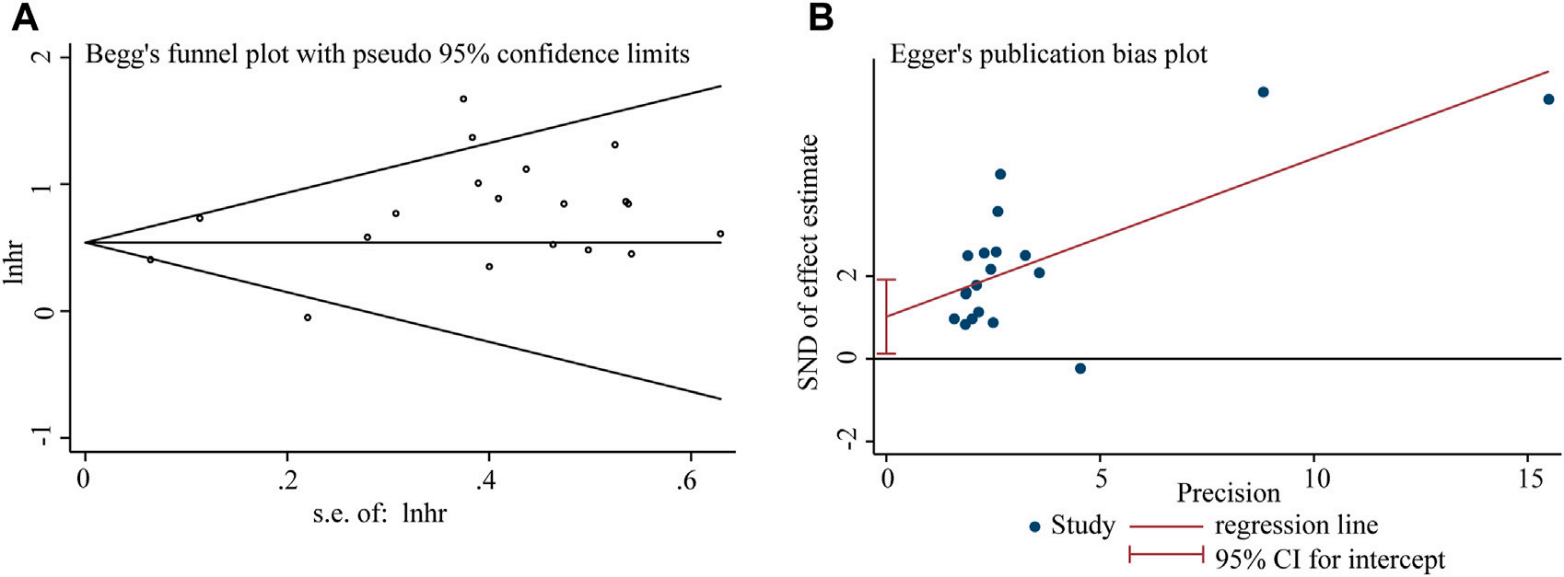
Publication bias analysis of overall survival (OS) data: **(A)** Begg’s funnel plot of overall survival (OS) in the published studies. **(B)** Egger’s publication bias plot of OS in the published studies. The X-axis represents the standard error, which is used to measure accuracy. The higher the standard error, the less accurate the test. HR is plotted on the Y-axis. The black dots indicate the impact of LncRNA BLACAT1 on the outcome.

**FIGURE 6:**
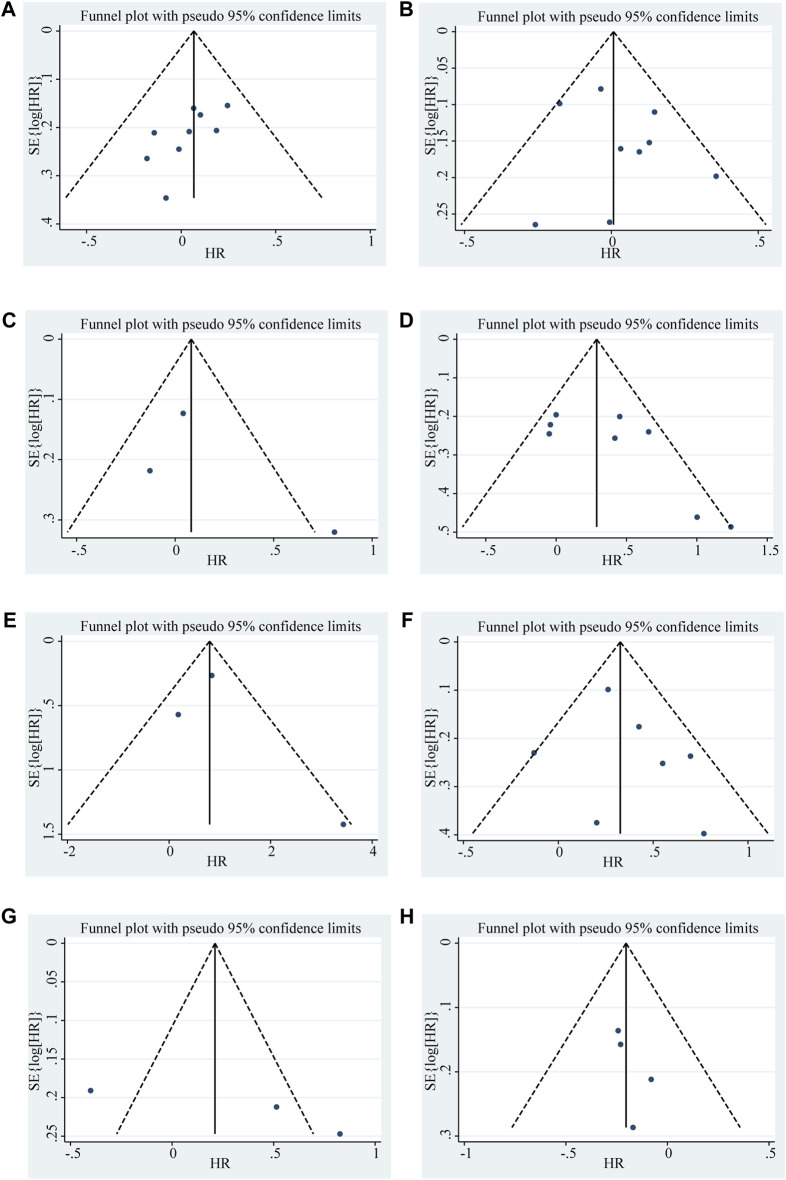
Funnel plots shows the association between the LncRNA BLACAT1 expression and clinicopathological characteristics: **(A)** age; **(B)** gender; **(C)** smoking; **(D)** lymph node metastasis; **(E)** distant metastasis; **(F)** clinical stage; **(G)** tumor size; **(H)** differentiation status. The Y-axis represents the standard error, which is used to measure accuracy. The higher the standard error, the less accurate the test. HR is plotted on the X-axis. The black dots indicate the impact of LncRNA BLACAT1 on the outcome.

Begg’s funnel plot and Egger’s test were utilized to evaluate publication bias in the current meta-analysis. The result of Begg’s test showed the absence of significant publication bias. The shape of the funnel plot was also symmetrically inverted funnels (Figure 5). Besides, funnel plots demonstrated that there was no obvious publication bias in clinicopathological characteristics (Figure 6).

### Sensitivity analysis

**FIGURE 7:**
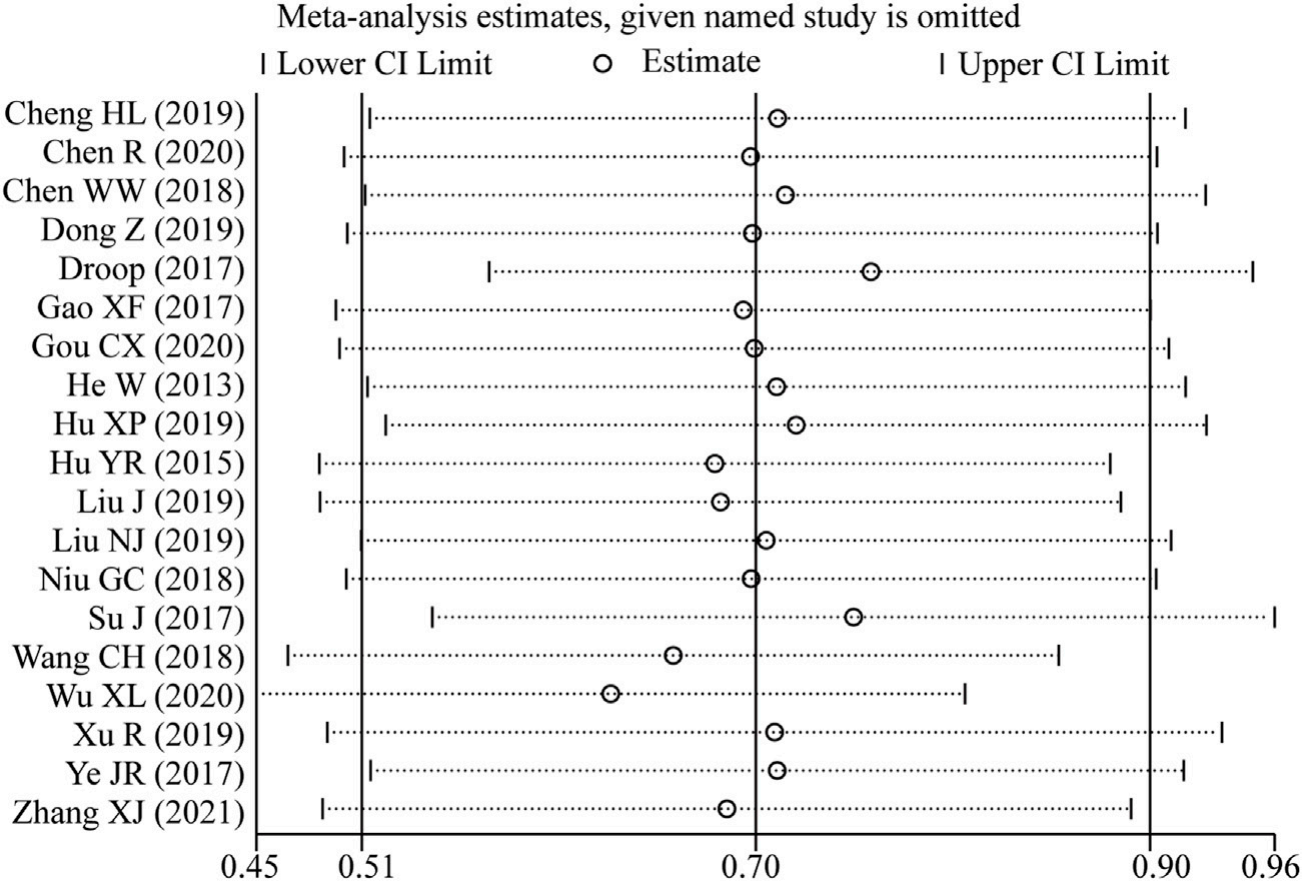
Sensitivity analysis of the pooled HRs of LncRNA BLACAT1 expression and overall survival (OS).

Sensitivity analysis was conducted by excluding one study every time which indicated that the pooled HRs were not significantly affected by the removal of any single study (Figure 7).

## Discussion

Cancer remains a major issue in healthcare and poses significant threats to human health (Siegel et al., 2019). Cancer metastasis, the process involving the dissemination of cancer cells from a primary lesion to distal organs, is the major cause of treatment failure and mortality in individuals with malignant tumors. It is considered as a multi-scale phenomenon, including molecular signaling networks, protein-protein interactions, metabolism, and cell-ECM interactions (Jia et al., 2022). Moreover, cancer lack of effective prognostic, metastasis, and diagnosis biomarkers, is primarily due to tumor heterogeneity (Khatib et al., 2020). Therefore, cancer metastasis has continued to confound researchers due to its complex, and not entirely predictable, pathological trajectory (Dudjak, 1992; Suhail et al., 2019). Currently, the precise metastasis mechanisms of human cancer are still unclear. Increasing evidence suggests that LncRNAs play an essential role in all stages of carcinogenesis and metastasis (Weidle et al., 2017), thus, it is considered as one of the most valuable biological markers (Soudyab et al., 2016; Deng et al., 2017; Aalijahan and Ghorbian, 2019; Galamb et al., 2019). LncRNA BLACAT1 has been demonstrated to be significantly upregulated in various cancers, including gastric cancer, cervical cancer (CC), breast cancer, and bladder cancer, etc. (Wang et al., 2018; Wu et al., 2018; Hu et al., 2019; Chen et al., 2020; Wang et al., 2020). The aberrant expression of LncRNA BLACAT1 resulted in a variety of oncogenic processes, including apoptosis, proliferation, cell cycle arrest, migration, etc. Thus, LncRNA BLACAT1 serves as an oncogene and plays critical roles in various biological and pathological processes. Wang et al. identified that the knock-down of LncRNA BLACAT1 significantly suppressed CC cell proliferation, migration, and invasion, while high LncRNA BLACAT1 expression served as a poor prognostic factor for CC patients (Wang et al., 2018). Another study indicated that LncRNA BLACAT1 overexpression could promote cervical cancer by downregulating miR-424 and miR-143 (Cheng et al., 2020). As for ovarian cancer, LncRNA BLACAT1 knockdown suppressed proliferation, migration, and invasion by suppressing the Wnt/β-catenin signaling in ovarian cancer cells (Yang et al., 2020). In colorectal cancer, high LncRNA BLACAT1 expression contributed to the poor overall survival rate and tumor progression by miR-519d-3p/CREB1 axis (Chen et al., 2020). These studies demonstrated that BLACAT1 plays a crucial role in tumor development and progression, and inspired us to investigate the correlation between LncRNA BLACAT1 expression level and cancer prognosis. In this meta-analysis, we comprehensively evaluate the relationship between BLACAT1 expression and prognosis and clinicopathological characteristics of tumors. The results illustrated that increased LncRNA BLACAT1 expression was significantly associated with unfavorable OS (HR: 2.02, 95% CI: 1.66–2.46) and PFS (HR: 2.424, 95% CI: 1.827–3.020), and similar results were obtained in further subgroup analysis. In addition, our results indicated that elevated level of LncRNA BLACAT1 was significantly related to positive lymph node metastasis, advanced clinical stage (*p* < 0.00001), and poor differentiation status, while no significant correlations were observed in age, gender, smoking, tumor size and distant metastasis. Furthermore, funnel plot and sensitivity analysis results revealed that our conclusion was reliable and robust. Taken together, these results demonstrated that LncRNA BLACAT1 could be applied as a novel predictor for prognosis in various tumors.

Nevertheless, several limitations in this study should be noted. First, the number of included studies and sample sizes were relatively small, especially for specific types of cancer, which might reduce the stringency of our conclusion. Second, there was no consensus on the cut-off value to define high lncRNA BLACAT1 expression and low lncRNA BLACAT1 expression. The included patients were divided into two groups based on the mean or median of BLACAT1 expression. Third, HRs with 95% CIs were extracted indirectly from survival curves rather than being directly obtained from the original studies, which might inevitably cause possible deviations; Fourth, most of the studies were from China, which limits the generalizability of our results. Thus, our results may only apply to the Chinese population. Despite some limitations mentioned above, our study confirmed the unfavorable role of elevated LncRNA BLACAT1 expression in malignant tumors and inspired researchers to further investigate the underlying mechanisms.

The use of biomarkers to predict therapeutic response is fundamental in precision medicine, thus more high-quality multicenter studies are required to further clarify the significance of LncRNA BLACAT1 in future cancer prognosis. There are several points to consider in this respect. First, the cut-off value should be demonstrated and unified to divide the high/low expression of biomarkers. Second, HRs with 95% CIs should be presented in the form of specific figures to avoid possible deviations. Third, further large-scale studies are needed in different populations. Fourth, the effect of covariates (age, gender, histological type, TNM stage, smoking, and so on) on outcomes should be considered.

In this study, we concluded that patients with high LncRNA BLACAT1 expression had shorter OS, suggesting that high levels of the LncRNA BLACAT1 may be detrimental to cancer patients. Moreover, when compared to patients with low expression, those with high LncRNA BLACAT1 expression tended to have an advanced clinical stage, earlier lymph node metastasis, and poorer differentiation status. All these results suggested that elevated LncRNA BLACAT1 expression may act as an oncogene factor and induce a worse prognosis in various cancers.

## Conclusion

In summary, this study suggested that elevated LncRNA BLACAT1 expression was negatively associated with OS, PFS, tumor differentiation, clinical stage, and lymph node metastasis. Therefore, LncRNA BLACAT1 may serve as a promising prognostic biomarker for cancer. Considering the limitations of this study, large-scale and multicenter studies will be needed to confirm these results and validate their clinical significance.

## Data Availability

The original contributions presented in the study are included in the article/Supplementary Material, further inquiries can be directed to the corresponding author.
