# Supplementary material for: The prognostic significance of LncRNA BLACAT1 overexpression in various tumors: a meta-analysis
Source: Front Genet. 2024 Mar 27;15:1362420. doi: 10.3389/fgene.2024.1362420 (PMC11004358; doi:10.3389/fgene.2024.1362420)
Supplement: Supplementary file 1 [file DataSheet1.docx]

**Supplementary Table 1：The details of the search strategies.**

| Database | Search strategy |
| --- | --- |
| PUBMED | ((("rna, long noncoding"[MeSH Terms] OR ("rna"[All Fields] AND "long"[All Fields] AND "noncoding"[All Fields]) OR "long noncoding rna"[All Fields] OR ("long"[All Fields] AND "noncoding"[All Fields] AND "rna"[All Fields])) AND "BLACAT1"[All Fields]) OR (("lncrnas"[All Fields] OR "rna, long noncoding"[MeSH Terms] OR ("rna"[All Fields] AND "long"[All Fields] AND "noncoding"[All Fields]) OR "long noncoding rna"[All Fields] OR "lncrna"[All Fields]) AND "BLACAT1"[All Fields]) OR "BLACAT1"[All Fields] OR (("urinary bladder neoplasms"[MeSH Terms] OR ("urinary"[All Fields] AND "bladder"[All Fields] AND "neoplasms"[All Fields]) OR "urinary bladder neoplasms"[All Fields] OR ("bladder"[All Fields] AND "cancer"[All Fields]) OR "bladder cancer"[All Fields]) AND ("associate"[All Fields] OR "associated"[All Fields] OR "associates"[All Fields] OR "associating"[All Fields] OR "association"[MeSH Terms] OR "association"[All Fields] OR "associations"[All Fields]) AND ("transcript"[All Fields] OR "transcript s"[All Fields] OR "transcripted"[All Fields] OR "transcription, genetic"[MeSH Terms] OR ("transcription"[All Fields] AND "genetic"[All Fields]) OR "genetic transcription"[All Fields] OR "transcription"[All Fields] OR "transcriptional"[All Fields] OR "transcriptions"[All Fields] OR "transcriptive"[All Fields] OR "transcripts"[All Fields]) AND "1"[All Fields]) OR "linc-UBC1"[All Fields]) AND ("cancer s"[All Fields] OR "cancerated"[All Fields] OR "canceration"[All Fields] OR "cancerization"[All Fields] OR "cancerized"[All Fields] OR "cancerous"[All Fields] OR "neoplasms"[MeSH Terms] OR "neoplasms"[All Fields] OR "cancer"[All Fields] OR "cancers"[All Fields] OR ("cysts"[MeSH Terms] OR "cysts"[All Fields] OR "cyst"[All Fields] OR "neurofibroma"[MeSH Terms] OR "neurofibroma"[All Fields] OR "neurofibromas"[All Fields] OR "tumor s"[All Fields] OR "tumoral"[All Fields] OR "tumorous"[All Fields] OR "tumour"[All Fields] OR "neoplasms"[MeSH Terms] OR "neoplasms"[All Fields] OR "tumor"[All Fields] OR "tumour s"[All Fields] OR "tumoural"[All Fields] OR "tumourous"[All Fields] OR "tumours"[All Fields] OR "tumors"[All Fields]) OR ("carcinoma"[MeSH Terms] OR "carcinoma"[All Fields] OR "carcinomas"[All Fields] OR "carcinoma s"[All Fields]) OR ("neoplasm s"[All Fields] OR "neoplasms"[MeSH Terms] OR "neoplasms"[All Fields] OR "neoplasm"[All Fields]) OR ("neoplasms"[MeSH Terms] OR "neoplasms"[All Fields] OR "neoplasia"[All Fields] OR "neoplasias"[All Fields])) |
| Cochrane Library | ((cancer):ti,ab,kw OR (tumor):ti,ab,kw OR (carcinoma):ti,ab,kw OR (neoplasia):ti,ab,kw OR (neoplasm):ti,ab,kw OR MeSH descriptor: [neoplasia] in all MeSH products) AND ((long noncoding RNA BLACAT1):ti,ab,kw OR (BLACAT1):ti,ab,kw OR (lncRNA BLACAT1):ti,ab,kw OR (bladder cancer associated transcript 1):ti,ab,kw OR (BLACAT1):ti,ab,kw ) |
| EMBASE | (' cancer '/exp OR cancer.ti,kw,hw OR ' tumor '/exp OR' tumor.ti,kw,hw OR carcinoma.ti,kw,hw OR ' carcinoma '/exp OR ' neoplasm '/exp OR neoplasm.ti,kw,hw ' neoplasia '/exp OR neoplasia.ti,kw,hw) AND (' long noncoding RNA BLACAT1 '/exp OR long noncoding RNA BLACAT1.ti,kw,hw OR ' lncRNA BLACAT1'/exp OR lncRNA BLACAT1. ti,kw,hw OR ' BLACAT1 '/exp OR BLACAT1.ti,kw,hw' OR ' bladder cancer associated transcript 1 '/exp OR bladder cancer associated transcript 1. ti,kw,hw OR linc-UBC1'/exp OR linc-UBC1.ti,kw,hw) |
| Web of science | ((((TS=(cancer)) OR TS=(tumor )) OR TS=(carcinoma)) OR TS=(neoplasm)) OR TS=(neoplasia) OR ((((TS=(long noncoding RNA BLACAT1)) OR TS=(lncRNA BLACAT1)) OR TS=(BLACAT1)) OR TS=( bladder cancer associated transcript 1)) OR TS=(linc-UBC1) |
| CNKI | (FT= “cancer” OR FT =“tumor ” OR FT=“carcinoma” OR FT=“neoplasm” OR FT=“neoplasia” ) AND (FT=“long noncoding RNA BLACAT1” OR FT=“lncRNA BLACAT1” OR FT=“BLACAT1” OR FT =“bladder cancer associated transcript 1” OR FT=“linc-UBC1” ) |
| WanFang | (AF = “cancer” OR AF =“tumor ” OR AF=“carcinoma” OR AF=“neoplasm” OR AF=“neoplasia” ) AND (AF=“long noncoding RNA BLACAT1” OR AF=“lncRNA BLACAT1” OR AF=“BLACAT1” OR AF =“bladder cancer associated transcript 1” OR AF=“linc-UBC1” ) |
